# Impacts of Physical and Biological Processes on Spatial and Temporal Variability of Particulate Organic Carbon in the North Pacific Ocean during 2003–2017

**DOI:** 10.1038/s41598-019-53025-4

**Published:** 2019-11-11

**Authors:** Jun Yu, Xiujun Wang, Hang Fan, Rong-Hua Zhang

**Affiliations:** 1 College of Global Change and Earth System Science, Beijing Normal University, Beijing, China; 2 Institute of Oceanology, Chinese Academy of Sciences, Qingdao, 266071 Shandong China; 3 Qingdao National Laboratory for Marine Science and Technology, Qingdao, 266237 China; 4 University of Chinese Academy of Sciences, Beijing, 10029 China

**Keywords:** Carbon cycle, Carbon cycle, Marine biology

## Abstract

The North Pacific Ocean is a significant carbon sink region, but little is known about the dynamics of particulate organic carbon (POC) and the influences of physical and biological processes in this region at the basin scale. Here, we analysed high-resolution surface POC data derived from MODIS-Aqua during 2003–2017, together with satellite-derived sea surface chlorophyll and temperature (SST). There are large spatial and temporal variations in surface POC in the North Pacific. Surface POC is much lower in the subtropical region (<50 mg m^−3^) than in the subarctic region (>100 mg m^−3^), primarily resulting from the south-to-north variability in biological production. Our analyses show significant seasonal and interannual variability in surface POC. In particular, there is one peak in winter-spring in the western subtropical region and two peaks in late spring and fall in the western subarctic region. Surface POC is positively correlated with chlorophyll (r = ~1) and negatively correlated with SST (*r* = ~−0.45, P < 0.001) south of 45°N, indicating the strong influence of physically driven biological activity on the temporal variability of POC in the subtropical region. There is a significantly positive but relatively lower correlation coefficient (0.6–0.8) between POC and chlorophyll and an overall non-significantly positive correlation between POC and SST north of 45°N, reflecting the reduction in the POC standing stock due to the fast sinking of large particles. The climate modes of the Pacific Decadal Oscillation, El Niño–Southern Oscillation and North Pacific Gyre Oscillation have large impacts on POC in various seasons in the subtropical region and weak influences in the subarctic region. Surface POC was anomalously high after 2013 (increased by ~15%) across the basin, which might be the result of complex interactions of physical and biological processes associated with an anomalous warming event (the Blob).

## Introduction

Particulate organic carbon (POC) is an important component in the oceanic carbon cycle. However, there are limited analyses on the dynamics of POC in the North Pacific, a region with significant carbon sinks^1^. Small-scale field studies have shown large spatial variability in the North Pacific. For example, there are much higher POC levels in the subarctic region than in the subtropical region of the northwest Pacific^2^ and modestly higher POC values to the west than to the east in the subarctic region^3^. Similar spatial variability was also observed in the POC flux in the North Pacific, e.g., there were higher values of POC flux in the subarctic (increasing from east to west) than in the subtropical Pacific^3–5^. The variability of POC export in the North Pacific is largely attributed to living POC components (i.e., phytoplankton and zooplankton)^5–8^.

The North Pacific Ocean undergoes strong temporal changes in physical processes, with implications for biogeochemistry. For example, from 1961 to 2012, there were strong interannual to decadal variabilities in surface water nutrients, with decreasing trends in phosphate and silicate, that were associated with changes in horizontal advection and/or vertical mixing^9,10^. Studies have also shown a decreasing trend in chlorophyll and net community production from 1971 to 2000 in the surface waters of the Northwest Pacific^11,12^. The outcomes of these earlier studies suggest that there may be large temporal variability in the surface POC of the North Pacific. However, the analyses addressing this issue are limited^13^; thus, little is known about the temporal variability of POC and its underlying mechanisms.

A number of studies have demonstrated that the biogeochemical changes in the North Pacific are associated with climate modes, such as the El Niño–Southern Oscillation (ENSO), Pacific Decadal Oscillation (PDO) and North Pacific Gyre Oscillation (NPGO)^10,14–16^. For example, a study showed that phytoplankton production in the eastern North Pacific is weakened during El Niño events due to the weakening or diminishing of upwelling^17^. There is also evidence that surface chlorophyll and nutrients are phase-locked with PDO in the North Pacific^10,18^. In addition, Di Lorenzo *et al*.^16^ reported that NPGO has played a dominant role in regulating surface salinity, nutrients and chlorophyll at the decadal timescale in the Northeast Pacific. These findings imply that climate modes have impacts on the temporal variability of the surface POC in the North Pacific Ocean.

Anomalous transient warm conditions (the Blob) have occurred in the surface water of the Northeast Pacific since late 2013^19^. Such unusual warming conditions exert broad impacts on marine ecosystems and biogeochemical processes^20,21^. While significant changes in the abundance and composition of phytoplankton have been observed in the northeast Pacific, the biological responses during the Blob are different between nitrate-limited and iron-limited regions^21^. Ultimately, warming and biological changes have various effects on POC dynamics in the upper water of the northeast Pacific, with implications for other parts of the North Pacific. However, the large-scale responses of POC in the North Pacific during the Blob are unknown.

Previous studies have indicated that there are large spatial and temporal variabilities in the surface POC of the North Pacific that are influenced by physical and biological processes. Our understanding of POC dynamics can be enhanced by satellite ocean colour observations with the recent development of POC algorithms^22^ that have shown overall satisfactory performances^23–26^. Here, we use high-resolution POC data derived from MODIS-Aqua over the period of 2003–2017. To examine the influences of physical and biological processes, we also utilize high-resolution datasets of remotely sensed sea surface temperature (SST) and chlorophyll. For example, we analyse the variability of the POC:chlorophyll (POC:Chl) ratio to explore the influence of biological processes on the POC composition. The objectives of this study are to evaluate the spatial and interannual variability of surface POC in the North Pacific, to analyse the different influences of physical and biological processes and to explore the impacts of major climate modes and the Blob on the dynamics of POC across the basin.

## Regional Setting

**Figure 1:**
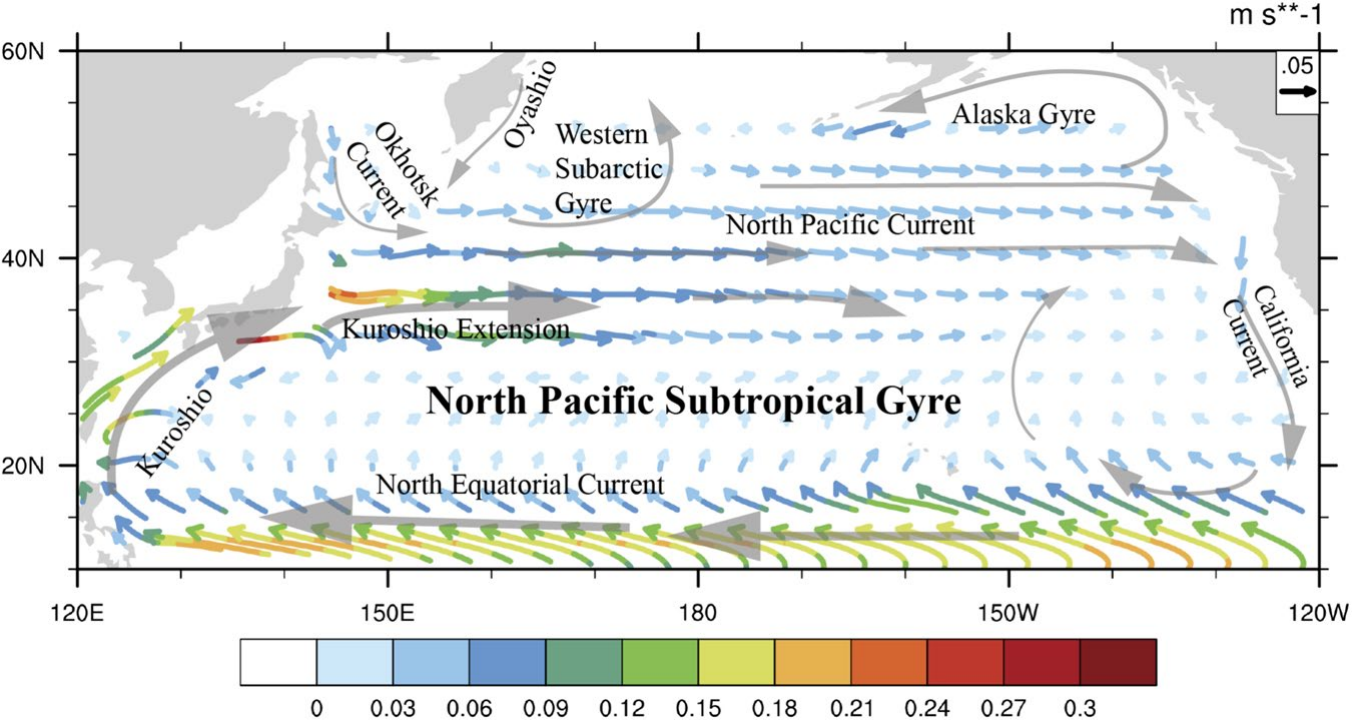
General circulation in the North Pacific showing the North Equatorial Current, Kuroshio, Kuroshio Extension, California Current, Alaska Gyre and Western Subarctic Gyre. The coloured arrows denote the climatology of the surface ocean current velocity over the period of 2003–2017, using data from the European Centre for Medium-range Weather Forecasts Ocean Reanalysis System 4 (ORAS4, http://icdc.cen.uni-hamburg.de/thredds/aggregationOras4Catalog.html). This figure was generated using the NCAR Command Language (version 6.4.0) [Software]. (2017). Boulder, Colorado: UCAR/NCAR/CISL/TDD. 10.5065/D6WD3XH5.

The North Pacific Ocean consists of two distinctive regions: the subtropical region and the subarctic region (Fig. 1). The former can be represented by the North Pacific Subtropical Gyre (NPSG), which is influenced by two major currents: the North Pacific Current to the north and North Equatorial Current to the south^27^. In addition, there exists the Kuroshio in the west and the California Current (CC) in the east. The California Current is the predominant flow in the eastern portion of the NPSG^28^, which has weaker physical variability than the western portion of the NPSG^27^. Overall, the surface water in the NPSG is known as an “ocean desert” due to the conditions of strong stratification and severe nutrient depletion in the upper layer^29^.

The subarctic Pacific, which is known as a high nutrient low chlorophyll (HNLC) region, consists of two major cyclonic gyres: the Western Subarctic Gyre (WSG) to the west and the Alaska Gyre (AG) to the east. The WSG is fed by cold nutrient-rich coastal currents, such as the southward Oyashio and the eastward Okhotsk Current^30^, and has much deeper halocline and winter mixed layer depths (MLD) than the AG^15^. The AG begins near 48°N, 130°W from the bifurcation of the North Pacific Current, flows northwestward due to geostrophic forcing and has a strong, shallow halocline layer due to high precipitation, low evaporation, and freshwater inputs from land runoff^31^.

## Results

### Spatial distributions

**Figure 2:**
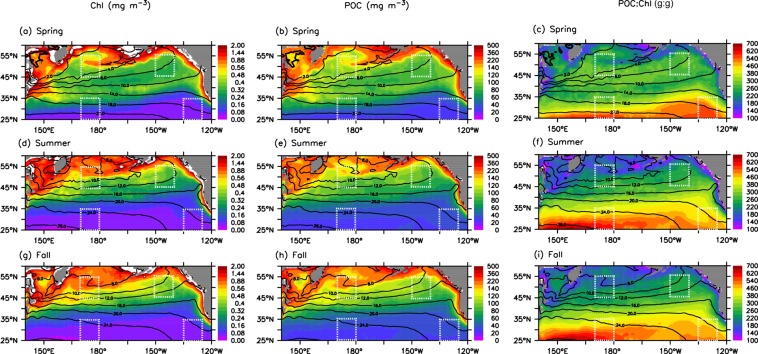
Spatial distribution of surface chlorophyll (Chl, left panel), POC (middle panel) and the POC:Chl ratio (right panel) in the (**a**–**c**) spring (Mar.–May), (**d**–**f**) summer (Jun.–Aug.) and (**g**–**i**) fall (Sep.–Nov.) over the period of 2003–2017. Superimposed black lines denote the climatology (2003–2017) of SST (°C) in the different seasons. Squares outlined by dashed white lines indicate the four studied areas (eastern/western subarctic and subtropical regions). This and the following figures were generated using Ferret v7.3, a product of NOAA’s Pacific Marine Environmental Laboratory (information is available at http://ferret.pmel.noaa.gov/Ferret/).

The climatology (2003–2017) of chlorophyll, POC and the POC:Chl ratio together with SST, are presented in Fig. 2. The winter season is not shown because there is a considerable amount of missing data at high latitudes (north of 50°N) due to cloud cover. Overall, the spatial variability of both chlorophyll and POC is large, with a clear south-to-north increase. For example, the POC level increases by a factor of two from the 20 °C isotherm (<50 mg m^−3^) to the 10 °C isotherm. Apart from the meridional variation, there is a pronounced zonal variability of POC in the open ocean north of the 15 °C isotherm, i.e., POC increases from the east (~100 mg m^−3^) to the west (~150 mg m^−3^). Furthermore, both POC and chlorophyll concentrations are significantly highest along coastal regions and in the Bering Sea and Okhotsk Sea. Despite the similarity between the spatial distributions of POC and chlorophyll, there is a large spatial variability in the POC:Chl ratio, with a significantly higher ratio (300–700 g:g) in the subtropical region but much lower ratio (~200 g:g) in the northern region, coastal areas and the continental margins. Overall, the spatial variability of the POC:Chl ratio is smaller in spring than in summer and fall.

### Seasonal variability

**Figure 3:**
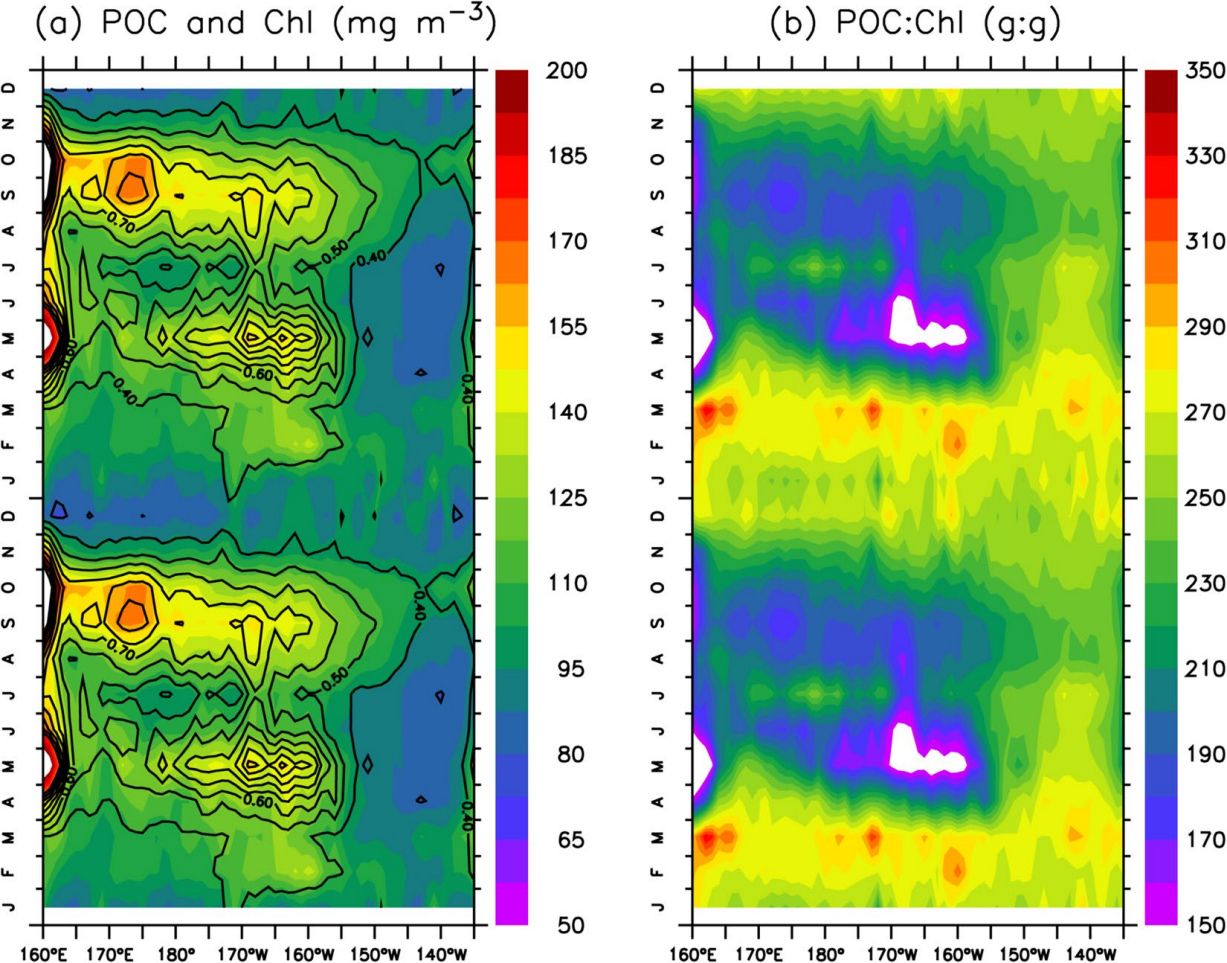
Seasonal climatology (with a repetition of another year) of (**a**) surface POC (coloured contours) and chlorophyll (black lines) and (**b**) the POC:Chl ratio over 45°N-55°N.

To examine the seasonal variability of chlorophyll and POC in the subarctic and subtropical regions, we focus on two representative latitude bands (i.e., 45–55°N and 25–35°N), analyse the seasonal mean and seasonal variability (coefficient of variation, CV) and compare the differences between the west and the east (Table 1). Overall, both chlorophyll and POC show modest seasonality in the subarctic region, with two peaks (i.e., in late spring and fall) to the west of 155°W **(**Fig. 3a**)**. Clearly, the seasonality of chlorophyll is stronger in the west (33% for CV) than in the east (10% for CV) (Table 1). POC shows a similar pattern, with larger CV in the west (19%) than in the east (6.7%). The eastern subarctic Pacific has small ranges of both chlorophyll (0.33~0.4 mg m^−3^) and POC (89–98 mg m^−3^), and as a result, the POC:Chl ratio (257–276 g:g) has a narrow range during all four seasons (Table 1). The POC:Chl ratio in the west is lower in summer-fall (~200 g:g) than in winter-spring (~270 g:g, Fig. 3b).

**Table 1:** Seasonal mean, standard deviation (s.d.), seasonal variability (CV: coefficient of variability) and statistical difference between east and west (pvalue) of the surface chlorophyll (mg m^−3^), POC (mg m^−3^) and POC:Chl ratio (g:g) in the four studied areas (from Fig. 2) in the seasons of spring, summer, fall and winter.

Area		45–55°N			25–35°N		
		170–180°E	150–140°W	Differ. (P value)	170–180°E	135–125°W	Differ. (P value)
		Mean (s.d.)	Mean (s.d.)		Mean (s.d.)	Mean (s.d.)	
Chl (mg m^−3^)	Spr.	0.47 (0.13)	0.33 (0.046)	<0.001	0.16 (0.048)	0.071 (0.016)	<0.001
	Sum.	0.56 (0.24)	0.36 (0.11)	<0.001	0.066 (0.012)	0.077 (0.0096)	<0.001
	Fall	0.68 (0.23)	0.40 (0.088)	<0.001	0.068 (0.019)	0.077 (0.011)	<0.01
	Winter	0.35 (0.052)	0.36 (0.073)	>0.05	0.16 (0.023)	0.11 (0.016)	<0.001
	CV	33%	10%		48%	22%	
POC (mg m^−3^)	Spr.	114 (19)	89 (11)	<0.001	51 (11)	32 (3.6)	<0.001
	Sum.	111 (22)	89 (13)	<0.001	31 (2.9)	34 (2.3)	<0.001
	Fall	136 (32)	98 (16)	<0.001	31 (4.3)	34 (2.5)	<0.001
	Winter	93 (15)	95 (20)	>0.05	53 (5.3)	42 (3.6)	<0.001
	CV	19%	6.7%		29%	12%	
POC: Chl (g:g)	Spr.	264 (25)	276 (19)	<0.01	414 (43)	508 (42)	<0.001
	Sum.	238 (26)	268 (21)	<0.001	512 (38)	462 (27)	<0.001
	Fall	219 (26)	257 (18)	<0.001	509 (54)	464 (30)	<0.001
	Winter	267 (16)	267 (18)	>0.05	365 (23)	402 (23)	<0.001
	CV	9.9%	3.2%		16%	9.4%

**Figure 4:**
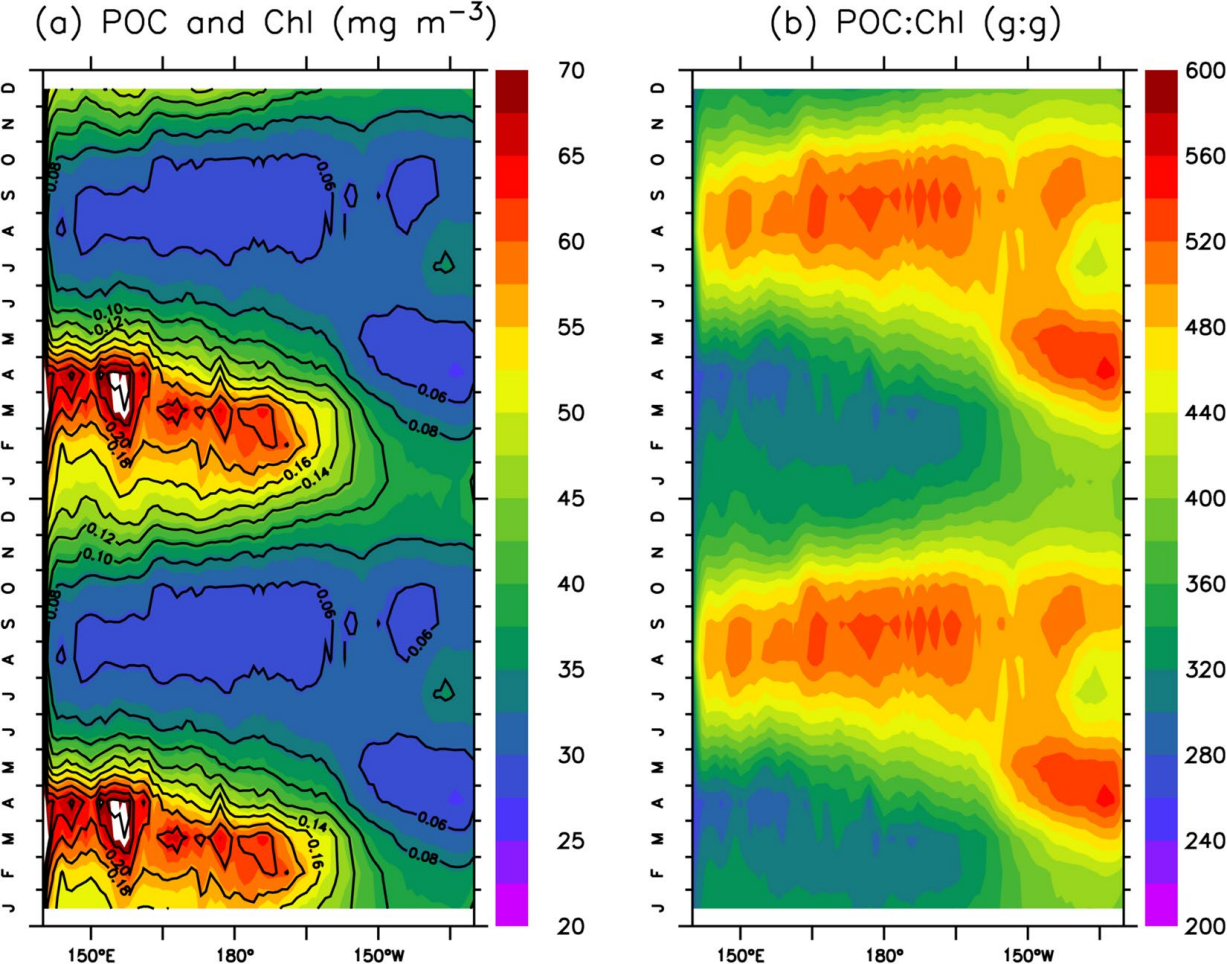
Same as Fig. 3, except over the subtropical region (25°N-35°N). Note that the scales in (**a**,**b**) are different from those in Fig. 3.

For the subtropical band, the seasonal variabilities of chlorophyll and POC are more pronounced (Fig. 4a), with higher CVs in this region than those in the subarctic region although the POC and chlorophyll concentrations are much lower in the former than in the latter (Table 1). Seasonal variability is also stronger in the west than in the east for both POC (CV = 29% vs. 12%) and chlorophyll (CV = 48% vs. 22%) in the subtropical region. Over the seasonal cycle, the western section has higher POC values (~50 mg m^−3^) in winter-spring than in summer-fall (~31 mg m^−3^) (Table 1). In contrast, the POC:Chl ratio in the west is lower (~400 g:g) in winter-spring than in summer-fall (~500 g:g) (Fig. 4b). The seasonal variability of the POC:Chl ratio is less pronounced in the eastern subtropical Pacific than in the western subtropical Pacific (Table 1), with narrow ranges for POC (30–40 mg m^−3^) and the POC:Chl ratio (400–500 g:g).

### Interannual variability

**Figure 5:**
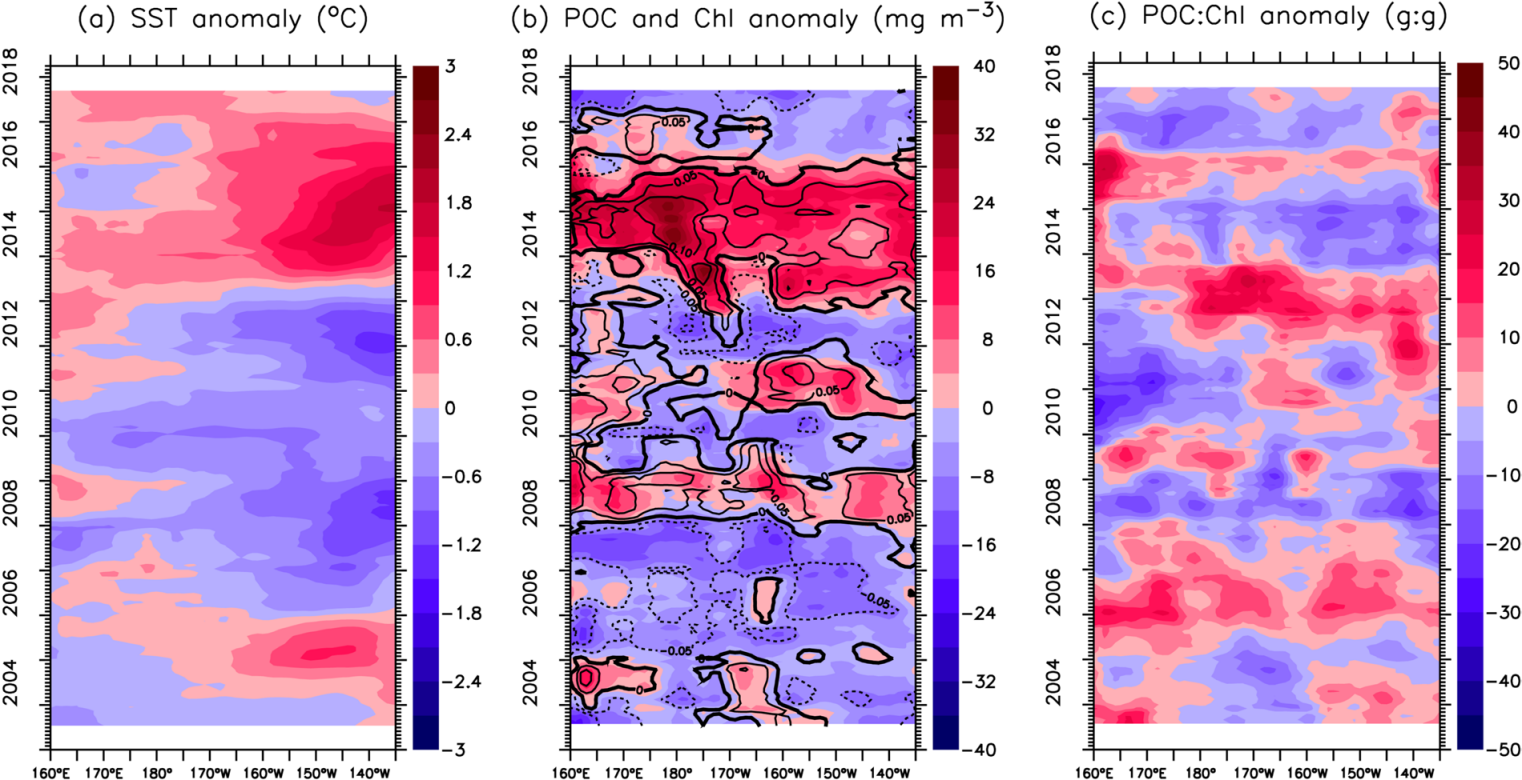
Time-longitude contours of anomalies for (**a**) SST, (**b**) POC (colour shade) and chlorophyll (black lines) and the (**c**) POC:Chl ratio over 45°N-55°N. The time-series are smoothed with a 13-month running mean.

We also evaluate the interannual variability of the SST, POC, chlorophyll and POC:Chl ratio over the two bands. Figure 5a illustrates that the SST variation is much larger in the east (with anomalies varying from −1 °C to +1.5 °C) than in the west (from −0.6 °C to +0.4 °C) in the subarctic band (45–55°N). In particular, the SST was clearly warmer during 2003–2005 and 2013–2016 than during 2006–2012 in the majority of the basin. Surface POC and chlorophyll have similar interannual variability (Fig. 5b), with high positive anomalies (10–20% increase in both chlorophyll and POC) during 2013–2015 under >2 °C warmer conditions. However, the temporal variations in POC and chlorophyll are different from those in SST prior to 2012, showing negative anomalies during 2005–2007 but positive anomalies during 2008. Unlike SST and POC, the POC:Chl ratio has no clear interannual variability (Fig. 5c), with positive anomalies (~30 g:g) in 2005/2006, 2012/2013 and 2015/2016 and negative anomalies (10–20 g:g) in 2008 and 2014.

**Figure 6:**
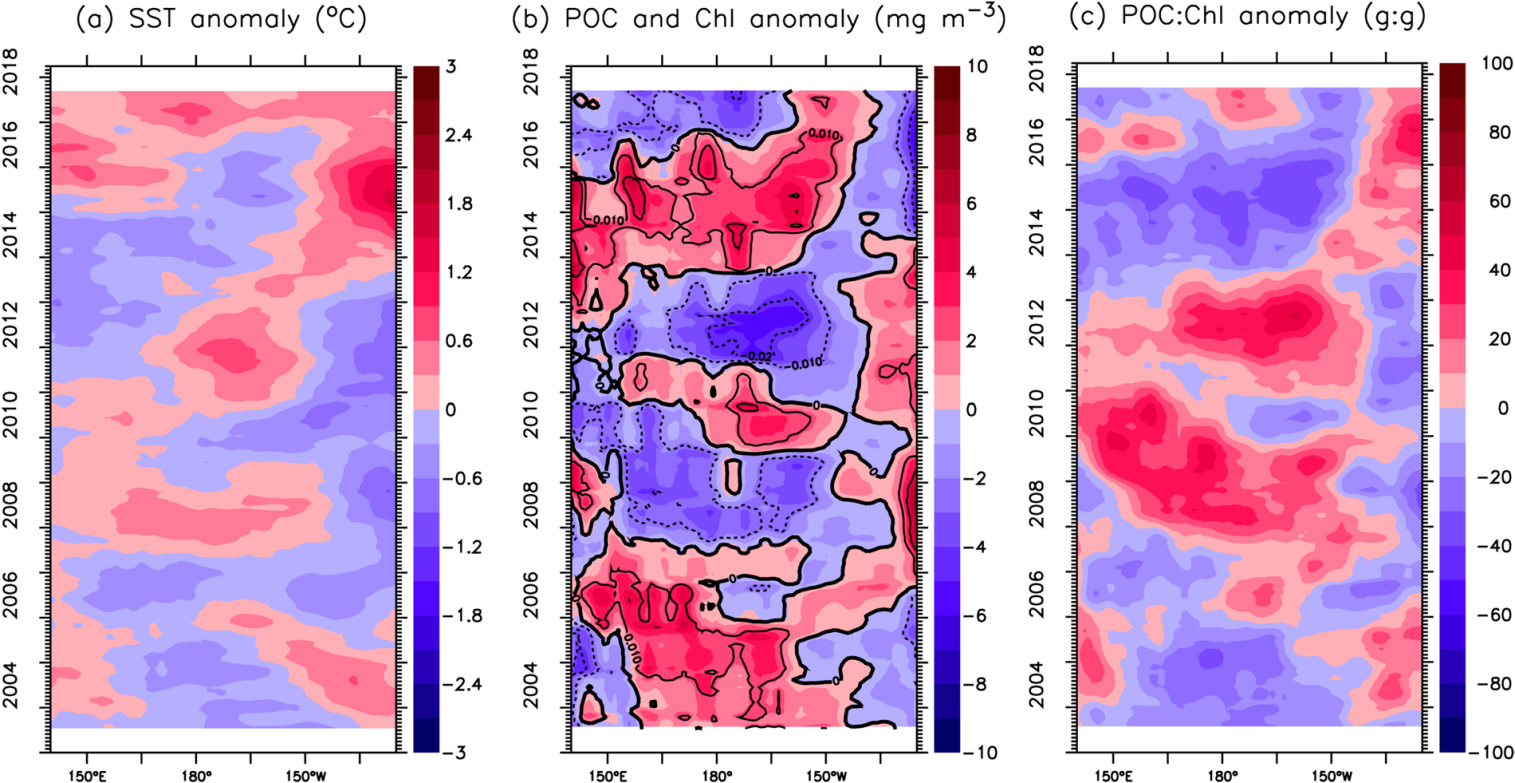
Same as Fig. 5, except over the subtropical region (25°N-35°N). Note that the scales in (**b**), (**c**) are different from those in Fig. 5.

There are clear differences in the SST anomalies between the west and the east in the subtropical region (25–35°N), e.g., there are negative anomalies during 2011–2014 in the west and during 2006–2012 in the east (Fig. 6a). Seemingly, there is an eastward propagation of positive SST anomalies, starting in 2008/9 along 150°E and reaching 130°W in 2015. Overall, the POC and chlorophyll anomalies are almost opposite to the SST anomalies, i.e., higher POC (chlorophyll) values corresponded to colder SSTs (Fig. 6b). However, the POC:Chl ratio shows a similar interannual variation as the SST anomaly (Fig. 6c) but has some degree of time lag in the west. Almost opposite to the POC anomaly, the highest POC:Chl ratio is found during 2008–2010 and 2012–2013, and the lowest POC:Chl ratio is found during 2014–2015.

## Discussion

### Spatial variability

An earlier study showed large spatial variability in primary production in the North Pacific^32^, i.e., an overall increase from the south to the north and an increasing trend from the east to the west, similar to the spatial pattern of POC (Fig. 2). The high production in the subarctic region is due to nutrient-rich conditions^17,29,31^. The zonal variability of primary production in the subarctic Pacific is suspected to be largely attributed to iron availability, i.e., iron is more available in the WSG than in the AG^30,33^.

The standing stock of POC is a balance between production and loss processes (e.g., the export of POC). There is evidence of more large-sized phytoplankton species (e.g., diatoms) in the subarctic region than in the subtropical region^32,34^. In general, particles generated from large phytoplankton and their pellets have relatively fast sinking velocities^4,34–37^, which can reduce the POC standing stock in the surface water, causing a relatively small POC:Chl ratio. Indeed, a field study showed a relatively small POC:Chl ratio (160~170 g:g) in the western subarctic region^38^. Overall, the observed POC:Chl ratios are close to the value (~200 g:g) representing the predominance of local biological activities in POC production^39,40^, indicating that biological processes are largely responsible for the higher levels of surface POC observed in the subarctic region.

The subtropical region reveals much lower levels of POC (<50 mg m^−3^), which are results of lower biological production^32^. A few studies indicate that chlorophyll variability in the subtropical Pacific is mainly driven by physiology, i.e., the small phytoplankton in this region have a high phytoplankton carbon to chlorophyll (C:Chl) ratio (150–250 g:g in the subtropical region, compared with 50–100 g:g for larger phytoplankton in the subarctic region) associated with low growth rates due to nitrate limitation and light acclimation^41–44^. Such a high C:Chl ratio in phytoplankton can be partly responsible for the increased POC:Chl ratios (300–600 g:g) in the subtropical region. On the other hand, the high POC:Chl ratios can also be attributed to the slow downward flux of POC in the surface water due to the relatively small sizes of phytoplankton and their pellets suspending in the surface water of the subtropical region^4,5,35^.

### Seasonal variability

**Figure 7:**
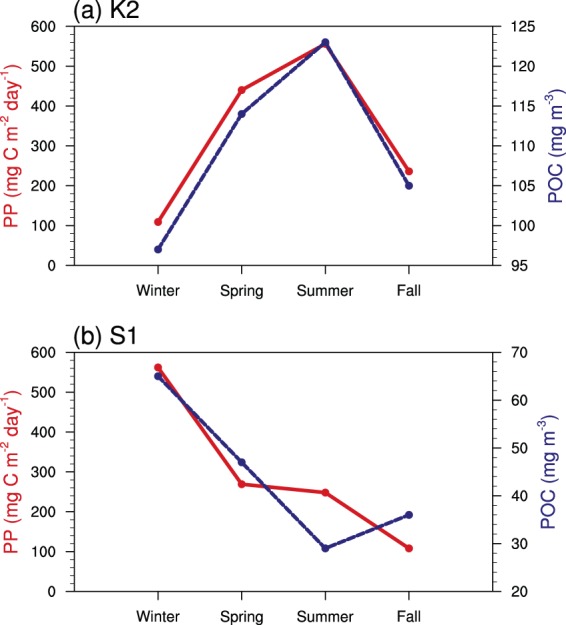
Comparisons of the seasonal variation between MODIS-derived POC (blue line, mg m^−3^) and *in situ* observations of primary production (PP, red line, mg C m^−2^ day^−1^) at (**a**) station K2 (160°E, 47°N) over 2005 to 2013 and at (**b**) station S1 (145°E, 30°N) over 2010 to 2013. The seasonal averages of *in situ* PP at K2 and S1 are from Sasai *et al*.^49^.

Previous studies showed a clear seasonality in satellite-derived primary production in the North Pacific, i.e., higher values in summer than in winter in the subarctic Pacific but higher values in winter than in summer in the subtropical Pacific^32,43^. Notably, this seasonality is similar to the seasonality of the satellite-derived POC in our study (Figs 3 and 4). Furthermore, we found similar seasonal variation between *in situ* primary production and satellite-derived POC in the northwest Pacific (Fig. 7). The agreement in the seasonality of POC and primary production indicates that biological production is largely responsible for the seasonal variability of POC.

The seasonality of POC is stronger to the west than to the east in the subarctic Pacific (Table 1). An earlier study showed much stronger seasonal variation of primary productivity in the western subarctic Pacific than in the eastern subarctic Pacific^45^, which was due to the larger seasonal variation in the iron supply in the west than in the east^30,46^. Apart from biological regulation, the relatively high POC:Chl ratio in winter in the west may indicate a supply of detrital POC, since there is evidence that more Okhotsk seawater flows into the Northwest Pacific through the southern Kuril Straits during the winter season^47^. On the other hand, the higher winter POC:Chl ratio is also probably associated with the predominance of small phytoplankton due to the low temperature and light limitation^48^.

The POC in the subtropical Pacific shows remarkable seasonal variability, with a pattern opposite to that of the POC:Chl ratio, i.e., a lower POC and higher POC:Chl ratio occur in summer-fall than in winter-spring. An earlier study demonstrated that the highest phytoplankton C:Chl ratios in this region typically occur in summer and fall^41^, suggesting that, apart from reductions in biological production, there might be further reductions in chlorophyll due to physiological responses. Based on the seasonal increases in POC (~20 mg m^−3^) and chlorophyll (~0.1 mg m^−3^) from summer-fall to winter-spring, we found that the change in the POC:Chl ratio is rather low (~200 g:g), indicating that biological processes are largely responsible for the seasonal increase in POC.

### Interannual variability

**Figure 8:**
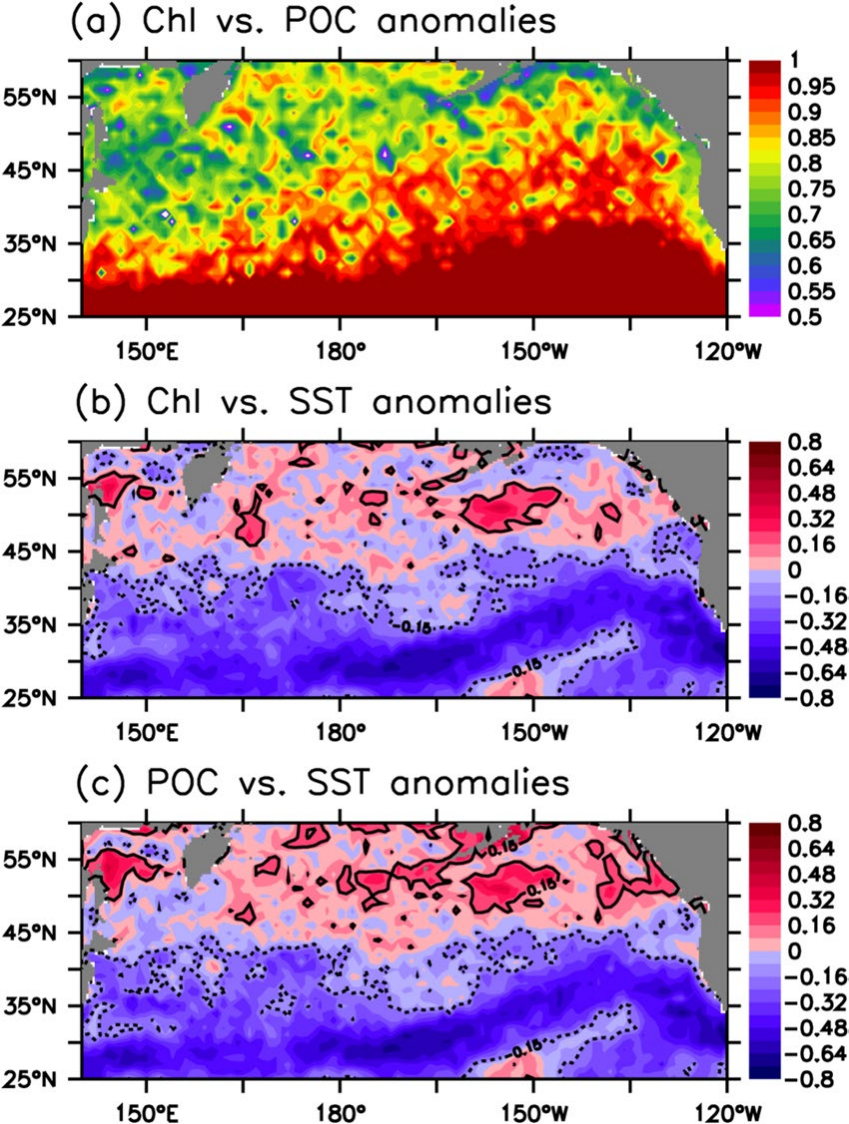
Spatial variations in the correlation coefficients (*r*) between the anomalies of (**a**) POC and chlorophyll, (**b**) chlorophyll and SST and (**c**) POC and SST over the period of 2013–2017. Superimposed solid (dashed) contours denote significant positive (negative) *r* values (0.15).

Our analyses show a significantly positive correlation (averaged *r* = ~0.75, P < 0.001) between the anomalies of chlorophyll and POC (Fig. 8a), suggesting that the interannual variability of POC in the North Pacific is primarily linked with biological processes. Interestingly, the correlation coefficient is smaller (*r* = 0.6~0.8) in the productive regions (e.g., subarctic region, coastal areas and marginal seas) than in the oligotrophic subtropical region (*r* = ~1), indicating that other processes influence POC variability. Indeed, there is evidence that large particles sink relatively fast in the subarctic region, particularly during seasons of high productivity^4,35^, which can cause a reduction in the POC standing stock in the surface water, thus altering its relationship with chlorophyll. There is a significantly negative correlation (averaged *r* = ~–0.45, P < 0.001) between chlorophyll/POC and SST south of 45°N (Fig. 8b,c), implying that physical processes play a large role in regulating the interannual variability of primary production and POC. Warmer SSTs reflect weaker vertical mixing, which causes poor nutrient conditions, lower phytoplankton production^49,50^ and thus less POC.

The relationship of POC with SST varies in the subarctic region, with a positive correlation in the majority of the basin. In general, rising temperatures and improved light availability (due to shoaling MLD) are beneficial for phytoplankton growth at high latitudes^38,50,51^. However, the overall non-significantly positive correlation between POC and SST and the relatively weak correlation between POC and chlorophyll in the subarctic region indicate that changes in the POC stock may not reflect changes in biological activity. First, the POC standing stock in the surface water can be reduced by downward export of large POC in the subarctic region^4,35^. Second, current systems can transport high-POC waters from nearshore to offshore and open ocean regions^52,53^, which is largely influenced by both local (e.g., winds and fresh water fluxes) and remote (e.g., basin-scale circulation) forcings^54^. These processes may have individual change patterns over an interannual time scale, thus altering the relationship between POC and biological production.

### Impacts of climate forcings

**Figure 9:**
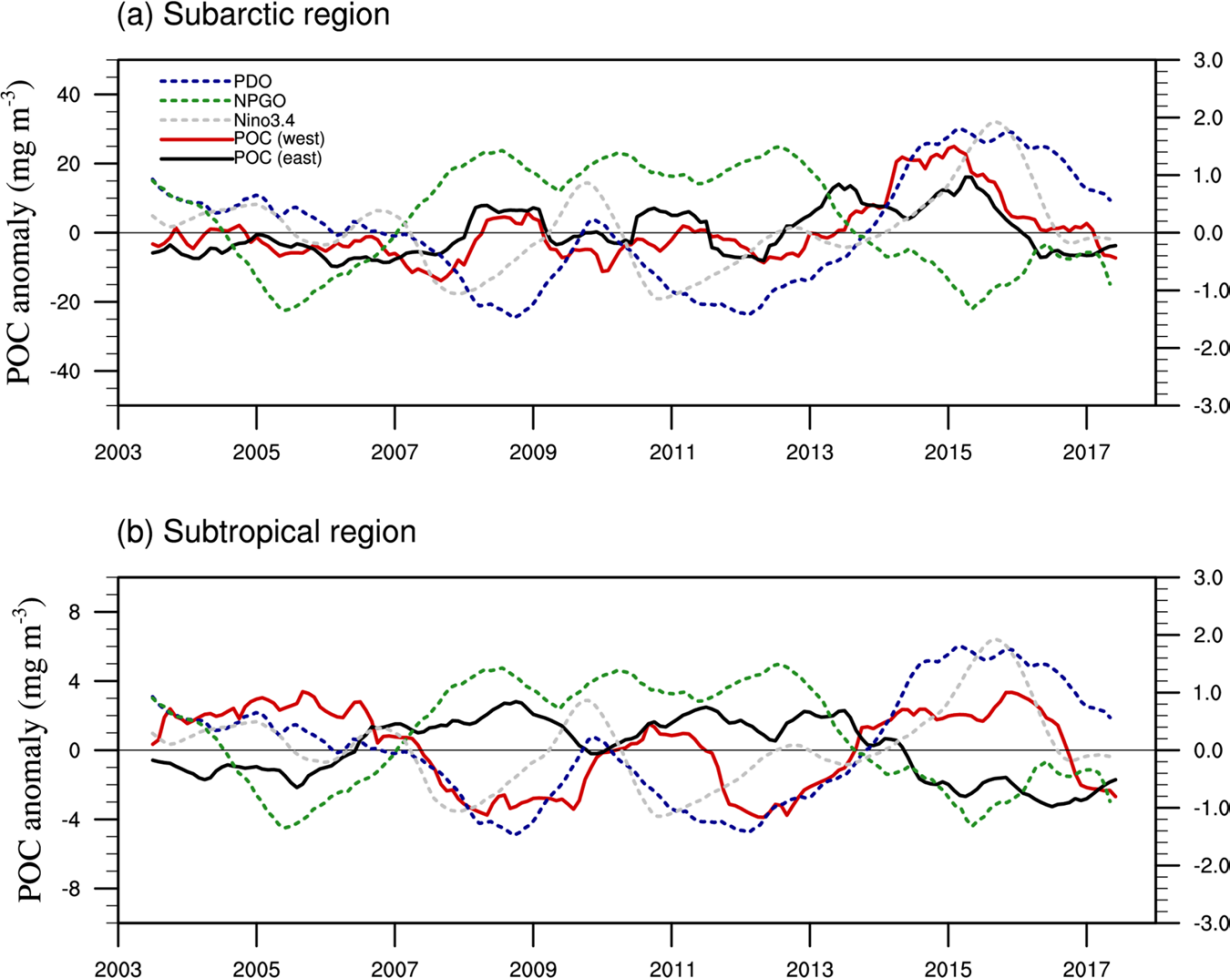
Time series of the indices of PDO (blue), NPGO (green), and Niño3.4 (grey) and POC anomalies in the west (red) and east (black) of (**a**) the subarctic region (170°E-180°E, 45°N-55°N and 150°W-140°W, 45°N-55°N) and (**b**) subtropical region (170°E-180°E, 25°N-35°N and 135°W-125°W, 25°N-35°N). The scales of POC in (**b**) are different from those in (**a**) (smaller). All time-series are smoothed with a 13-month running mean.

It is generally recognized that physical and biogeochemical processes are largely influenced by climate forcings in the North Pacific^10,18,55^. Here, we further analyse the interannual variability of POC and its relationships with climate modes (ENSO, PDO, NPGO) in different regions. Figure 9 illustrates large differences in the POC-climate mode relationship between the subarctic and subtropical regions. Based on the correlation coefficient estimated with data over 2003–2013 (excluding the influence of the Blob after 2014), it is clear that the POC anomaly showed no relationship with the climate modes in the subarctic region (Table 2). Similarly, an earlier analysis based on SeaWiFS data (over 1997–2010) revealed that chlorophyll had an insignificant correlation with PDO and NPGO in the subarctic Pacific^56^. However, a more recent study using SeaWiFS/MODIS data over a longer period (1997–2012) showed various responses in different seasons, e.g., increased chlorophyll in winter but decreased chlorophyll in spring and summer during the positive phase of PDO in the western subarctic Pacific^18^. We analysed the relationship between chlorophyll and PDO/NPGO for respective seasons, but there were no statistically significant correlations (data not shown) in the subarctic Pacific for any seasons during 2003–2013.

**Table 2:** Correlation coefficients (*r*) between the POC anomalies and PDO, Niño3.4 and NPGO indices for each season and for all-seasons in the four studied areas over the period of 2003–2013.

		Winter (n = 33)	Spring (n = 33)	Summer (n = 33)	Fall (n = 33)	All-seasons (n = 132)
Northwest	PDO	−0.27	0.17	−0.18	−0.24	−0.11
	Niño3.4	−0.03	0.08	0.03	−0.07	−0.01
	NPGO	0.04	−0.1	0.02	0.15	0.03
Northeast	PDO	−0.16	−0.06	−0.15	−0.08	−0.11
	Niño3.4	−0.16	−0.10	0.03	−0.07	−0.1
	NPGO	0.06	0.09	0.17	0.05	0.08
Southwest	PDO	0.08	0.55***	0.62***	0.54**	0.38***
	Niño3.4	0.03	0.2	−0.06	0.39*	0.17*
	NPGO	−0.56***	−0.24	−0.51**	−0.47**	−0.36***
Southeast	PDO	−0.61***	−0.68***	−0.60***	−0.49**	−0.56***
	Niño3.4	−0.35*	−0.48**	−0.42*	−0.28	−0.33***
	NPGO	0.03	0.36*	0.47**	0.27	0.23**

*p < 0.05; **p < 0.01; ***p < 0.001.

On the other hand, POC had significant correlations with climate modes in most seasons in the subtropical region (Table 2). As a result, POC was significantly correlated with climate modes in all seasons, and the strongest correlation was with PDO in both the west (*r* = 0.38, P < 0.001) and the east (*r* = −0.56, P < 0.001). It appears that the correlation of POC was stronger with NPGO than with ENSO in the west but stronger with ENSO in the east. Clearly, there were opposite POC-climate relationships between the west and the east, e.g., POC was higher in the west but lower in the east during the warm phases of PDO and ENSO (see Fig. 9b), suggesting different physical and/or biological responses in these regions. There is evidence of stronger westerly wind in the west but northward advection of warm air in the east during the warm phases of PDO and ENSO^57^, resulting in higher levels of nutrients in the west than in the east^9,10^. Indeed, field studies showed lower rates of primary production in the eastern subtropical Pacific during the warm phases of PDO^58^ and ENSO^58–60^, as a consequence of increased stratification and reduced nutrient mixing^61^. Similarly, the POC-NPGO relationship was also opposite between the west and the east (Table 2), which can be ascribed to downwelling-favourable conditions of the Subtropical Gyre and upwelling-favourable conditions of the California Current during the positive phases of NPGO^57,62^.

**Figure 10:**
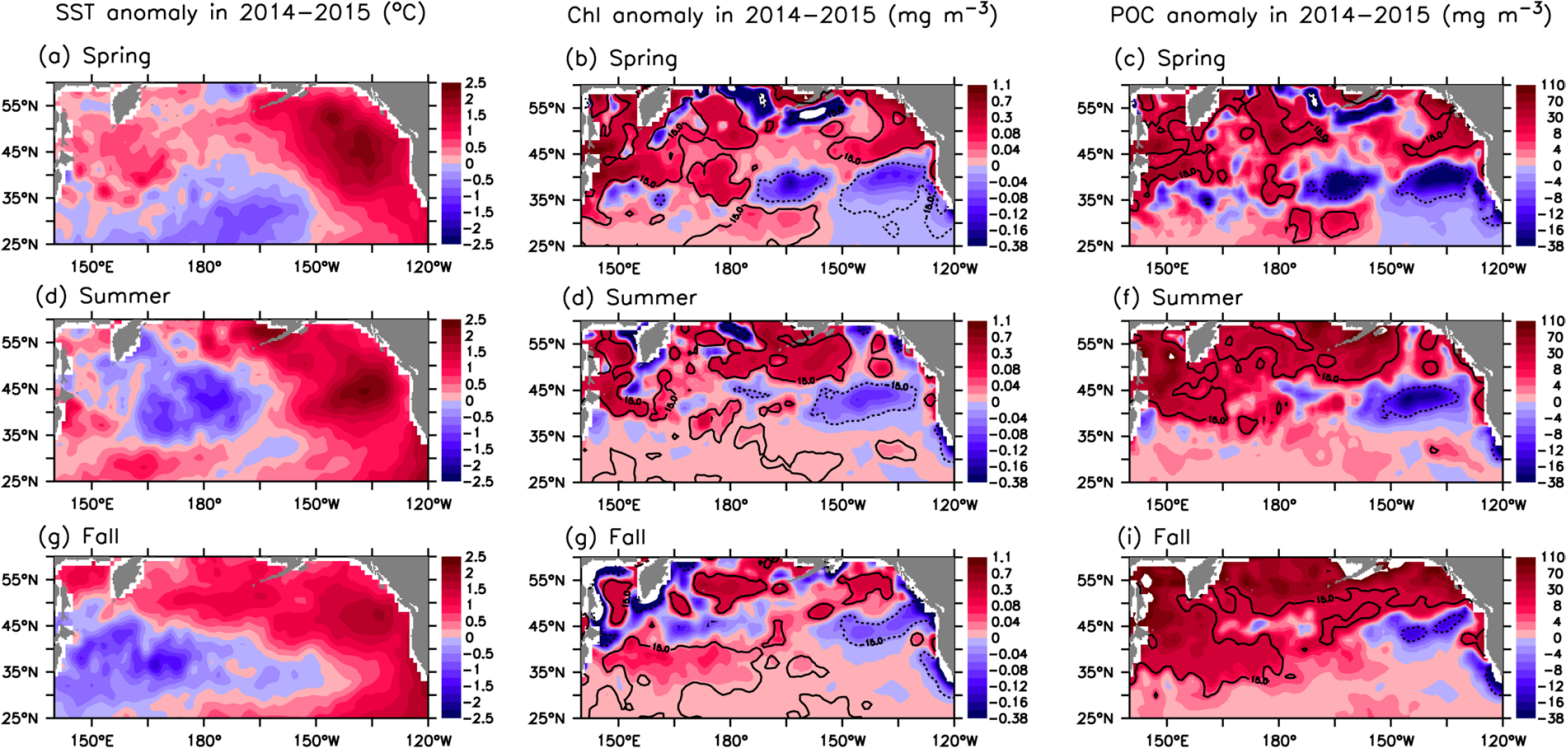
Spatial variation in the 2014–2015 anomalies (deviation from the means over 2003–2017) of SST (left panel), chlorophyll (middle panel) and POC (right panel) in (**a–c**) spring (Mar.–May), (**d–f**) summer (Jun.–Aug.) and (**g–i**) fall (Sep.–Nov.). Superimposed solid (dashed) lines denote a 15% increase (decrease). All maps are smoothed spatially by a 3° × 3° low-pass filter.

A number of studies have reported that the record-high SST anomaly (the Blob) triggered an unprecedented bloom of warm-water planktonic species in the coastal regions from southern California to the Aleutian Islands during 2014–2015^20,63,64^, and our analyses show an overall increase in both chlorophyll and POC during the same period in the north Pacific (Figs 5b and 6b). Here, we further assess the basin-scale anomalies of chlorophyll and POC (i.e., deviation from the mean over 2003–2017) during this extreme warming event. Given the fact of inconsistent responses of chlorophyll to climate forcing in different seasons^18^, we compare the anomalies of chlorophyll and POC with the SST anomaly in the main seasons (spring, summer and fall) (Fig. 10). Clearly, SST shows an overall increase in the subarctic region, except for in summer in the west, but a decrease in some parts of the subtropical region (Fig. 10a,d,g). The positive SST anomaly in 2014–2015, mainly occurring in the Northeast Pacific, has been described elsewhere^19,65,66^. Clearly, the spatial patterns of warming and the changes in chlorophyll and POC are not similar. On the other hand, there is an overall similarity between the spatial patterns of the chlorophyll and POC anomalies, except for in fall. In particular, both chlorophyll and POC increase in most sections of the subarctic region in spring and summer but decrease in the eastern subtropical region in spring. The increase in POC is greatest in fall in terms of magnitude and spatial coverage, with a large area showing a >15% increase, while the change in chlorophyll in fall shows no similarity.

There is evidence of strengthened northwesterly winds in the western-central part of the subtropical region in winter-spring during the Blob^65^, which could increase the nutrient supply due to enhanced vertical mixing and horizontal transport^67,68^, thus increasing the primary production and POC level. For the subarctic region, while the overall increase in chlorophyll may be associated with warming that can enhance biological production in this region^51,69^, the insignificant correlation between chlorophyll and SST (Fig. 8b) indicates that there may be other mechanisms causing the enhanced biological activities and/or elevated POC. A field study reported increased phytoplankton biomass in the eastern subarctic region associated with the Blob, with a significant increase in chlorophyll (~100%, relative to 2013) during 2014, and it is speculated that this increase might result from improved light and nutrient conditions^21^. In addition, there is evidence of extreme sea ice melting event in the Okhotsk Sea in 2015^70^, which may supply iron into the surface water and thus enhance primary production in the subarctic region^71–73^. In addition, there might be increased secondary production through grazing in association with warming^74–76^, leading to an increase in POC in the subarctic region. Indeed, a recent study showed evidence of increased zooplankton biomass in the Gulf of Alaska during 2014–2015^77^. On the other hand, the overall increasing trend in POC from spring to fall (Fig. 10c,f,i) may reflect the accumulation of detrital POC through the food web following the spring phytoplankton bloom^78,79^. In summary, there may be complex physical processes associated with the Blob event, which could have impacts on biogeochemical processes and ecosystem dynamics, with implications for the sources/sinks of POC in the north Pacific. Therefore, process-based physical-ecosystem models are needed in future studies to quantify the key physical and biogeochemical processes that regulate the oceanic carbon cycle due to changes in environmental conditions.

## Conclusions

We analysed the spatial and temporal variability of surface POC in the North Pacific Ocean, together with surface chlorophyll and SST. Overall, the POC level varies by a factor of two from the subtropical to the subarctic region, i.e., there are much higher values of POC in the subarctic region (>100 mg m^−3^) than in the subtropical region (<50 mg m^−3^), similar to the spatial distributions of chlorophyll and primary production. However, the POC:Chl ratio is lower in the subarctic region (or when productivity is high), which might be partly attributed to the decreased phytoplankton C:Chl ratio and rapid sinking of large-size particles. The seasonality of POC manifests large meridional and zonal differences, with one peak in winter-spring in the western subtropical region and two peaks in late spring and fall in the western subarctic region. Surface POC has a significantly positive correlation with chlorophyll (*r* = ~1) and a negative correlation with SST (*r* = ~−0.45, P < 0.001) south of 45°N, indicating that physically driven biological activity is responsible for the temporal variability of POC in the subtropical region. There is also a significantly positive but lower correlation coefficient (0.6–0.8) between POC and chlorophyll and an overall non-significantly positive correlation between POC and SST north of 45°N, which reflects the reduction of the POC standing stock in the surface water due to the faster sinking of large particles. Our analyses demonstrate that the climate modes of PDO, ENSO and NPGO have large impacts on the POC dynamics in the subtropical region, showing opposite correlations between the west and the east. The Blob may have complex influences on physical and biological processes, leading to an increase in POC (by ~15%) during 2014–2015 in the majority of the basin.

## Data and Methods

### Satellite data descriptions

In this study, we used MODIS-Aqua-derived chlorophyll-a, POC and SST over the period of 2003–2017. The level 3 chlorophyll-a data were derived from the standard NASA algorithm, i.e., the OC3M band ratio algorithm from O’Reilly *et al*.^80^ merged with the colour index (CI) algorithm from Hu *et al*.^81^. The CI algorithm was a three-band reflectance (R_rs_) difference algorithm, using the difference between the R_rs_ in the green band and a reference formed linearly between R_rs_ in the blue and red bands, i.e.:

1 $$CI={R}_{rs}({\lambda }_{green})-[{R}_{rs}({\lambda }_{blue})+\frac{({\lambda }_{green}-{\lambda }_{blue})\times ({R}_{rs}({\lambda }_{red})-{R}_{rs}({\lambda }_{blue}))}{{\lambda }_{red}-{\lambda }_{blue}}]$$

where λ_blue,_ λ_green_ and λ_red_ represent the instrument-specific wavelengths closet to 443, 555 and 670 nm, respectively. The chlorophyll derived from the CI algorithm is defined as:

2 $${\log }_{10}(chl\_a)=191.659\times {\rm{CI}}-0.4909$$

The OC3M algorithm is a fourth-order polynomial relationship between the ratio of R_rs_ and chlorophyll-a, i.e.:

3 $${\log }_{10}(chl\_a)={a}_{0}+\mathop{\sum }\limits_{i=1}^{4}\,{a}_{i}{({\log }_{10}(\frac{{R}_{rs}({\lambda }_{blue})}{{R}_{rs}({\lambda }_{green})}))}^{i}$$

where a_0_-a_4_ are sensor-specific factors, which can be found in the algorithm description file of NASA (https://oceancolor.gsfc.nasa.gov/atbd/chlor_a/). The OC3M algorithm was used when the chlorophyll retrievals were above 0.2 mg m^−3^, and the CI algorithm was used for clearer water (chlorophyll < 0.15 mg m^−3^). In between these values, the CI and OC3M algorithms were both used by a weighted approach.

The level 3 POC data were based on the algorithm from Stramski *et al*.^22^, an empirical relationship derived from *in situ* measurements of POC and blue-to-green band ratios of spectral remotely sensed reflectances:

4 $$\begin{array}{c}POC={\rm{a}}\times {(\frac{{R}_{rs}({\lambda }_{blue})}{{R}_{rs}({\lambda }_{green})})}^{b}\end{array}$$

where a and b are set as 203.2 and −1.034, respectively.

### Match-up analysis

Satellite-derived chlorophyll product has been widely validated and showed quite good performance^26,82–84^. There were also some validations of satellite POC^23,24,26,85^. In particular, Swirgon and Stramska^23^ conducted a match-up analysis of *in situ* and satellite (SeaWiFS and MODIS Aqua) POC in the North Pacific over 1997–2012, which showed an overall good performance of satellite-derived POC. To further evaluate the quality of MODIS-Aqua-derived POC in the North Pacific over our study period, we carried out a match-up analysis by comparing *in situ* POC data in the surface water with satellite data during the period of 2003–2017. *In situ* POC data were obtained from the public databases of the SeaWiFS Bio-optical Archive and Storage System (SeaBASS, https://seabass.gsfc.nasa.gov/) and the Hawaii Ocean Time-series (HOT) programme (http://hahana.soest.hawaii.edu/hot/hot-dogs/interface.html) and the literature for the K2-S1 project in the Northwest Pacific^7,86,87^. Our comparison had a focus on the open ocean thus we didn’t use the *in situ* data from the coastal waters. In total, we found 759 *in situ* POC data points.

We downloaded the level 3 daily MODIS Aqua POC products (4 km spatial resolution) from https://oceandata.sci.gsfc.nasa.gov/MODIS-Aqua/Mapped/Daily/4km/. Satellite POC data were paired with *in situ* data with a spatial and temporal window^23,26,82^. The spatial window in our study was set as a 3 × 3 pixel box centred on the location of the *in situ* measurements. The box was only selected when at least 6 of 9 pixels were valid and the coefficient of variation was less than 15%. For the temporal window, we first tried the approach of Swirgon and Stramska’s match-up^23^, i.e., the time difference between the satellite passing time (13:30 P.M local time) and the *in situ* sampling time was less than 2 h, which yielded only 19 match-ups. Following a more recent validation work on satellite-derived chlorophyll and POC^26^, we applied a temporal window of 24 h and found 41 match-ups that were from 2003 to 2015.

**Figure 11:**
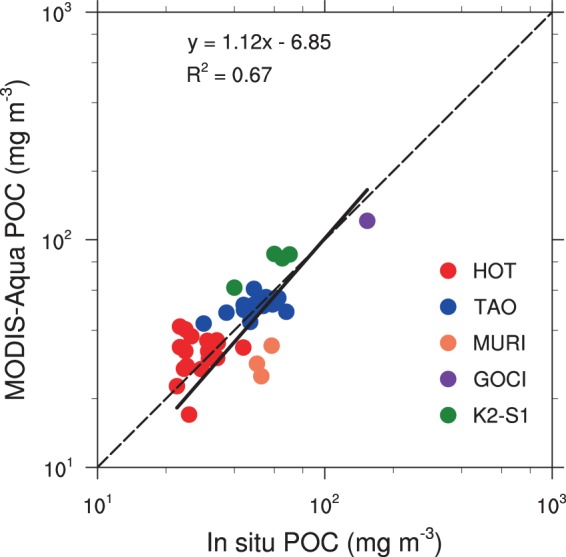
Scatterplot of MODIS Aqua-derived POC to *in situ* POC match-up in the North Pacific on logarithmic scale. The dashed line shows a 1:1 relationship, and the solid line is the best linear fit to the data. *In situ* POC data from the following experiments were used: the Hawaii Ocean Time-series (HOT, 22.75°N, 158°W), the Tropical Atmosphere Ocean project (TAO, 0~10°N, 140~120°W), the K2-S1 project (30–47°N, 140~160°E), the Multiple University Research Initiative project (MURI, 19~22°N, 159~155°W) and the Geostationary Ocean Colour Imager Validation Campaign (GOCI, 34.02°N, 126.23°E).

We adopted the Mode II reduced major axis regression approach following the previous validation studies^23,26,82^, accounting for the uncertainties in both *in situ* and satellite data, which showed a significantly good regression (R^2^ = 0.67, P < 0.001), with a slope of 1.12 (Fig. 11). The differences between *in situ* and satellite-derived POC were also evaluated by standard methods^82^, namely, the mean normalized bias (*P*_*bias*_), the root mean square error (RMSE) and the mean absolute percentage error (MPE), which were calculated as follows:

5 $$\begin{array}{c}{P}_{bias}=\frac{{\sum }_{i=1}^{N}\,({M}_{i}-{O}_{i})}{{\sum }_{i=1}^{N}\,{O}_{i}}\ast 100\end{array}$$

6 $$\begin{array}{c}RMSE={[\frac{1}{N}\mathop{\sum }\limits_{i=1}^{N}{({M}_{i}-{O}_{i})}^{2}]}^{1/2}\end{array}$$

7 $$\begin{array}{c}MPE=\frac{1}{N}\mathop{\sum }\limits_{i=1}^{N}\,|\frac{{M}_{i}-{O}_{i}}{{O}_{i}}|\ast 100\end{array}$$

where N is the number of match-ups (41), and *O*_*i*_ and *M*_*i*_ represent the values of *in situ* observations and MODIS POC, respectively. The evaluation showed an overall acceptable performance of MODIS-Aqua POC in the North Pacific (Fig. 11), with small values for P_bias_ (2.7%), RMSE (13.08 m^−3^) and MPE (22%), which indicated the reliability of MODIS-Aqua-derived POC for the analyses of large-scale spatial and temporal variabilities in the North Pacific.

### Time-series analyses

We obtained MODIS-Aqua monthly means of chlorophyll-a, POC and SST over the period of 2003–2017 with a 4 km spatial resolution from https://oceandata.sci.gsfc.nasa.gov/MODIS-Aqua/Mapped/Monthly/4km. We adopted linear interpolation from the nearest surrounding points to fill the missing data in the monthly MODIS POC and chlorophyll and re-gridded to the spatial resolution of 1° × 1°. We calculated anomalies by subtracting the climatological monthly mean (2003–2017) from monthly data. The Niño3.4 index, defined as the SST anomaly for the Niño3.4 region (i.e., 5°N-5°S, 120°−170°W), was obtained to represent the ENSO event^88^ (https://www.cpc.ncep.noaa.gov/data/indices). We also downloaded the PDO (http://research.jisao.washington.edu/pdo/) and NPGO (http://www.o3d.org/npgo/npgo.php) indices. The PDO and NPGO indices are defined as the first and the second corresponding principal components of SST anomalies and sea-surface height anomalies over the northeast Pacific (180°W–110°W; 25°N–62°N)^62^. Correlation analyses were carried out to examine the relationships between the POC anomalies and the anomalies of other variables or climate indices.

## Data Availability

The satellite data are available at https://oceandata.sci.gsfc.nasa.gov. The *in situ* data are available at https://seabass.gsfc.nasa.gov/ and http://hahana.soest.hawaii.edu/hot/hot-dogs/interface.html.
